# A technique for measuring anisotropy in atrial conduction to estimate conduction velocity and atrial fibre direction

**DOI:** 10.1016/j.compbiomed.2018.10.019

**Published:** 2019-01

**Authors:** Caroline H. Roney, John Whitaker, Iain Sim, Louisa O'Neill, Rahul K. Mukherjee, Orod Razeghi, Edward J. Vigmond, Matthew Wright, Mark D. O'Neill, Steven E. Williams, Steven A. Niederer

**Affiliations:** a School of Imaging Sciences and Biomedical Engineering, King's College London, London, United Kingdom; b LIRYC Electrophysiology and Heart Modeling Institute, Campus Xavier Arnozan, Avenue du Haut Lévêque, 33600, Pessac, France; c Univ. Bordeaux, IMB, UMR 5251, F-33400, Talence, France

**Keywords:** Conduction velocity, Anisotropy, Atrial fibres, Atrial fibrillation, Fibrosis

## Abstract

**Background:**

Cardiac conduction properties exhibit large variability, and affect patient-specific arrhythmia mechanisms. However, it is challenging to clinically measure conduction velocity (CV), anisotropy and fibre direction. Our aim is to develop a technique to estimate conduction anisotropy and fibre direction from clinically available electrical recordings.

**Methods:**

We developed and validated automated algorithms for estimating cardiac CV anisotropy, from any distribution of recording locations on the atrial surface. The first algorithm is for elliptical wavefront fitting to a single activation map (method 1), which works well close to the pacing location, but decreases in accuracy further from the pacing location (due to spatial heterogeneity in the conductivity and fibre fields). As such, we developed a second methodology for measuring local conduction anisotropy, using data from two or three activation maps (method 2: ellipse fitting to wavefront propagation velocity vectors from multiple activation maps).

**Results:**

Ellipse fitting to CV vectors from two activation maps (method 2) leads to an improved estimation of longitudinal and transverse CV compared to method 1, but fibre direction estimation is still relatively poor. Using three activation maps with method 2 provides accurate estimation, with approximately 70% of atrial fibres estimated within $20^{\circ }$. We applied the technique to clinical activation maps to demonstrate the presence of heterogeneous conduction anisotropy, and then tested the effects of this conduction anisotropy on predicted arrhythmia dynamics using computational simulation.

**Conclusions:**

We have developed novel algorithms for calculating CV and measuring the direction dependency of atrial activation to estimate atrial fibre direction, without the need for specialised pacing protocols, using clinically available electrical recordings.

## Highlights

• We developed a technique for estimating conduction anisotropy and fibre direction.
• To estimate anisotropy, we fit elliptical wavefront propagation to a single activation map.
• Using three activation maps provided accurate estimation of conduction anisotropy.
• We demonstrated heterogeneous conduction anisotropy in clinical recordings.
• Anisotropy affected predicted arrhythmia dynamics in computational simulation.

## 1 Introduction

Patient specific electrophysiology, anatomy and structure affect atrial fibrillation (AF) mechanisms. These features exhibit large variability between patients, and also change with AF progression. As such, determining each of their individual contributions to AF dynamics and sustaining mechanisms in an individual patient is both important and challenging. Changes that occur during AF that modify atrial conduction include down-regulation and lateralisation of connexins, deposition of collagen and interstitial fibrosis, as well as changes in atrial fibre direction, including fibre disarray [1,2]. Each of these factors affect the heterogeneity and anisotropy of atrial conduction. However, the quantitative relationship between fibrotic remodelling and longitudinal and transverse conduction velocity (CV), and the effects of each of these on AF in individual patients are unknown.

Measurements of the velocity and directional dependency of the propagation of the electrical signal across cardiac tissue can indicate properties of the underlying myocardium, where slower CV is thought to occur in diseased tissue [3]. Calculating CV and its anisotropy in clinical electrophysiology cases is a challenge, and there is currently no agreement on the best technique to quantify CV clinically [[4], [5], [6], [7], [8]]. Typically clinical measurements of conduction properties calculate the overall conduction speed and previous studies have found correlations between this measurement and other properties, including measures of atrial fibrosis from structural imaging data [9], electrogram amplitude and fractionation [10], and arrhythmia properties such as critical driver locations [11]. These varied and often weak correlations may represent the presence of distinct direction dependent changes in conduction [12], requiring the measurement of both longitudinal and transverse CV to show a clear relationship.

Previous studies have estimated longitudinal and transverse CV from a single activation map [13,14]. However, these methodologies require manual selection of longitudinal fiber direction, which is not feasible for analysing high-density global activation maps. We previously developed an automated technique for estimating CV and source location, assuming a planar or circular wavefront and constant CV [15], using recordings from an arbitrary arrangement of points. This algorithm may be applied to any multipolar catheter arrangement, provided the measuring point locations can be approximated locally as lying on plane.

Here we initially extend our algorithm to consider an elliptical wavefront of activation, to automatically provide estimates of both longitudinal and transverse CV from a single activation map (*method 1: elliptical wavefront fitting to a single activation map*). The algorithm works in cases of surface curvature by determining for each subset of recording points on the atrial surface a two-dimensional flattening that preserves geodesic distances between these surface points. This methodology performs well in the vicinity of the pacing location, but the accuracy decreases further from this location due to the effects of heterogeneities in the fibre field. As such, we develop a second methodology for estimating conduction anisotropy using ellipse fitting to planar estimates of CV measured from two or three pacing directions to estimate the longitudinal fibre direction, and longitudinal and transverse CV magnitudes (*method 2: ellipse fitting to wavefront propagation velocity vectors from multiple activation maps*). Our aim is to develop a technique that may be used to estimate conduction anisotropy and fibre direction from clinically available electrical recordings.

## 2 Methods

We initially describe a novel methodology for automatically estimating longitudinal and transverse CV by elliptical wavefront fitting to a single activation map using a methodology that incorporates surface curvature (method 1; Sections 2.1- 2.3); we then develop a technique for estimating fibre direction and anisotropy by ellipse fitting to wavefront propagation velocity vectors estimated from two or three activation maps (method 2; Section 2.4). Finally, we describe the clinical data (Section 2.5) and the simulation data (Section 2.6) used for testing the algorithms.

### 2.1 CV estimation: assuming a planar or circular wavefront

We previously developed a methodology for calculating the propagation CV assuming a planar or circular wavefront with isotropic conductivity measured at an arbitrary arrangement of points. For these two cases we quote the equations, and direct the interested reader to [15] for a derivation. We will then go on to derive the equation for an elliptical wavefront in Section 2.2 as might be observed on a homogeneous anisotropic plane.

**Fig. 1:**
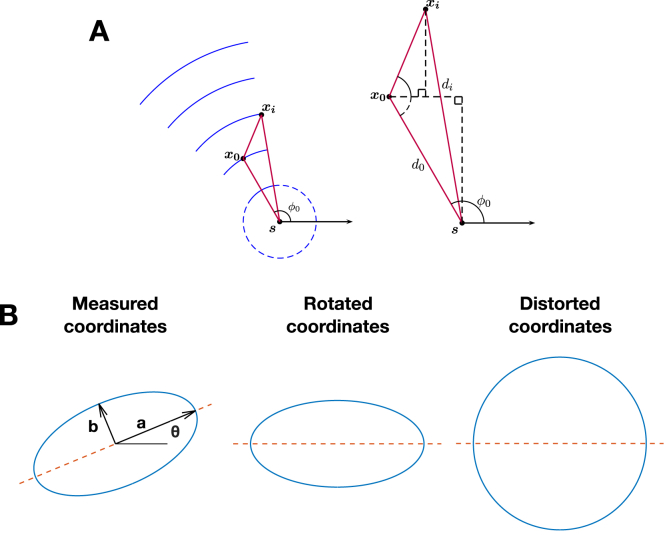
**Conduction velocity is estimated assuming planar, circular or elliptical wavefront propagation.** (A) Set-up considered for estimating the CV and source location of a circular wavefront, from Ref. [15] with permission. (B) An elliptical wavefront is mapped to a circular wavefront by first rotating by −θ and then scaling the *y*-axis, such that the circular wavefront algorithm can be applied.

Referring to Fig. 1 (A), we consider a wavefront that originates from an unknown source location $s=\left(s_{x},s_{y}\right)$ at an unknown time *T*, which propagates with unknown constant speed *v*. Our known recordings are at measuring locations $x_{i}=\left(x_{i},y_{i}\right)$, for *n* measuring points corresponding to i=0,…,n−1, ordered by activation time $t_{i}$. We then express our equations in terms of these parameters together with the following unknown parameters: $\phi_{0}$, the angle subtended at s by the *x*-axis and the earliest measuring point $x_{0}$, and the radius of curvature $d_{0}=||x_{0}-s||$, which represents the unknown distance from the source location s to the earliest measuring point $x_{0}$.

For planar wavefronts, the activation time of measuring location $x_{i}$ can be derived geometrically as

(1) $$t_{i}=\alpha_{0}+\alpha_{1}X_{i}+\alpha_{2}Y_{i},$$

where $X_{i}=x_{i}-x_{0}$, $Y_{i}=y_{i}-y_{0}$ are the differences in the coordinates to the first measuring points, and $\alpha ={\left(T,\;v^{-1}\text{cos}\phi_{0},\;v^{-1}\text{sin}\phi_{0}\right)}^{⊤}$. This is a linear least squares problem, for which the unknown parameters α are solved to give estimates for *v*, $\phi_{0}$ and *T*.

For circular wavefronts, the activation time of point $x_{i}$ can be derived to be

(2) $$t_{i}=T+\frac{1}{v}\sqrt{d_{0}^{2}+2\left(d_{0}\;\cos\phi_{0}X_{i}+d_{0}\;\sin\phi_{0}Y_{i}\right)+X_{i}^{2}+Y_{i}^{2}}.$$

Note that for this case, the source location is given by $s=x_{0}-\left(\begin{array}{c} d_{0}\;\cos\phi_{0}\\ d_{0}\;\sin\phi_{0} \end{array}\right)$.

To solve, we consider

(3) $$t_{i}=\beta_{0}+\beta_{1}\sqrt{\left(\beta_{2}^{2}+\beta_{3}^{2}\right)+2\left(\beta_{2}X_{i}+\beta_{3}Y_{i}\right)+X_{i}^{2}+Y_{i}^{2}},$$

where the coefficients $\beta ={\left(T,\;v^{-1},\;d_{0}\;\cos\phi_{0},\;d_{0}\;\sin\phi_{0}\right)}^{⊤}$.

Equation (3) is a non-linear least-squares problem in β, which can be solved by minimising $\sqrt{{\sum^{}}_{i=0}^{m-1}{\left(t_{i}-{\hat{t}}_{i}\right)}^{2}}$. It is solved using lsqnonlin in Matlab, with initial estimates for $\phi_{0}$ and *v* derived from the planar fit. The unknowns are then easily deduced from the values of β.

### 2.2 CV estimation: ellipse fitting to CV vectors from multiple activation maps (method 1)

For the case of elliptical wavefronts, we consider an equivalent set-up with measurements at known recording locations $x_{i}$, again ordered by activation time $t_{i}$, and the following unknowns: a source location s with activation time *T*, longitudinal and transverse velocity ($CV_{L}$ and $CV_{T}$ respectively), and ellipse long axis orientation *θ*. Our approach is to apply a linear transformation to map the elliptical wavefront to a circular wavefront, such that the transformed coordinates satisfy Equation (2). The necessary transformation *M* (see Fig. 1 (B)) consists of a rotation by −θ to align the ellipse long axis onto the *x*-axis, followed by a *y*-axis scaling to stretch the ellipse short axis to equal the long, that is:

(4) $$M=\left(\begin{array}{cc} 1 & 0\\ 0 & \frac{CV_{L}}{CV_{T}} \end{array}\right)\left(\begin{array}{cc} \cos\;\theta & \sin\;\theta \\ -\sin\;\theta & \cos\;\theta \end{array}\right).$$

Let ${\hat{d}}_{0}$, ${\hat{\phi }}_{0}$ be the distance and angle from the activation source to the first activation point after the transformation *M* is applied. Observe that the transformed $X_{i}$, $Y_{i}$ (i.e. ${\hat{X}}_{i}$ and ${\hat{Y}}_{i}$) are unknown functions of $CV_{L}$, $CV_{T}$ and *θ*:

(5) $${\hat{X}}_{i}=X_{i}\;\cos\;\theta +Y_{i}\;\sin\;\theta ,$$

(6) $${\hat{Y}}_{i}=\frac{CV_{L}}{CV_{T}}\left(Y_{i}\;\cos\;\theta -X_{i}\;\sin\;\theta \right).$$

Equation (2) then becomes:

(7) $$t_{i}=T+\frac{1}{CV_{L}}\sqrt{{\hat{d}}_{0}^{2}+2\left({\hat{d}}_{0}\;\cos{\hat{\phi }}_{0}{\hat{X}}_{i}+{\hat{d}}_{0}\;\sin{\hat{\phi }}_{0}{\hat{Y}}_{i}\right)+{\hat{X}}_{i}^{2}+{\hat{Y}}_{i}^{2}}.$$

Substituting for $\hat{X_{i}}$ and $\hat{Y_{i}}$ in terms of knowns $X_{i}$ and $Y_{i}$, we get:

(8) $$t_{i}=\gamma_{0}+\gamma_{1}{\left[\begin{array}{c} \left(\gamma_{2}^{2}+\gamma_{3}^{2}\right)+2\gamma_{2}\left(\sqrt{1-\gamma_{5}^{2}}X_{i}+\gamma_{5}Y_{i}\right)+2\gamma_{3}\left(\gamma_{4}\sqrt{1-\gamma_{5}^{2}}Y_{i}-\gamma_{4}\gamma_{5}X_{i}\right)\\ +{\left(\sqrt{1-\gamma_{5}^{2}}X_{i}+\gamma_{5}Y_{i}\right)}^{2}+{\left(\gamma_{4}\sqrt{1-\gamma_{5}^{2}}Y_{i}-\gamma_{4}\gamma_{5}X_{i}\right)}^{2} \end{array}\right]}^{\frac{1}{2}},$$

where the coefficients $\gamma ={\left(T,\;\frac{1}{CV_{L}},\;{\hat{d}}_{0}\;\cos{\hat{\phi }}_{0},\;{\hat{d}}_{0}\;\sin{\hat{\phi }}_{0},\;\frac{CV_{L}}{CV_{T}},\;\sin\;\theta \right)}^{⊤}$.

Upon numerically solving for *γ* and deducing $\hat{s}$, the original source location is found by applying the inverse transformation $M^{-1}$:

(9) $$s=\left(\begin{array}{cc} \cos\;\theta & -\sin\;\theta \\ \sin\;\theta & \cos\;\theta \end{array}\right)\left(\begin{array}{cc} 1 & 0\\ 0 & \frac{CV_{T}}{CV_{L}} \end{array}\right)\hat{s}.$$

### 2.3 Geodesic distances

We assume that the atrium is thin-walled with fibers running tangentially, and that it is transmurally homogeneous. CV calculations were performed on atrial surface meshes generated from an electro-anatomic mapping (EAM) system, or on simulation meshes downsampled to match the resolution of the EAM system meshes. For method 1, CV vectors were calculated for each element of the mesh using recording locations within an area of 1 cm × 1 cm around the element mid-point, to model recordings from electrodes on a multi-polar catheter, and provide a sufficient number of points for fitting. For method 2, a smaller area of 0.5 cm × 0.5 cm was used to calculate a more local planar CV (mean number of points included in the fit: 19.97 ± 0.41, range 12–20). These areas were determined in initial parameter sensitivity testing.

**Fig. 2:**
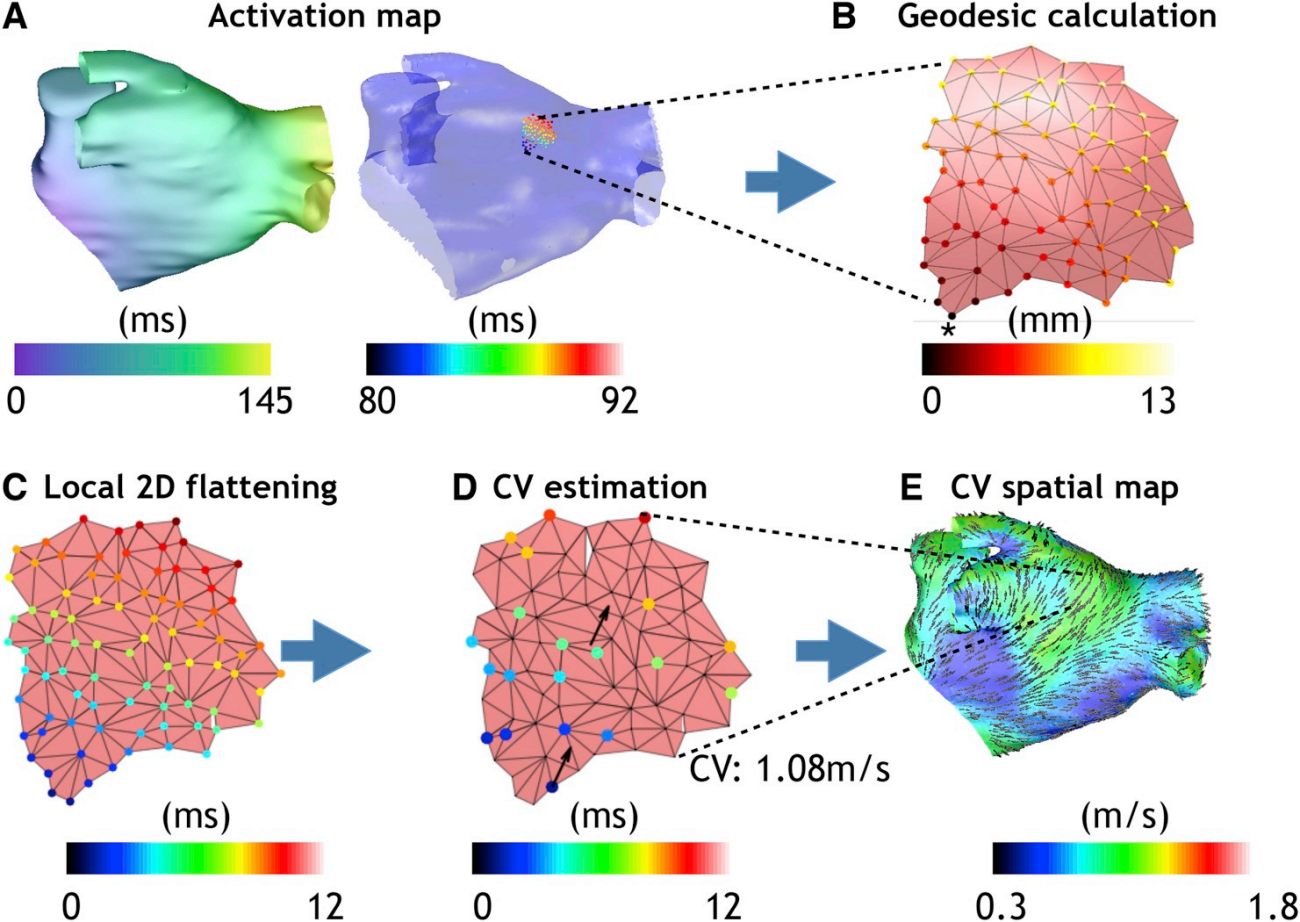
**Methodology for estimating CV on an atrial surface mesh.** (A) Select a local patch of tissue (1 cm × 1 cm) from an activation time map for analysis, centred around a measurement location. (B) Calculate geodesic distances on this patch of tissue. These are shown from an example vertex (indicated by the asterisk) to all other vertices, and then calculated with each vertex as a starting node to obtain a matrix of geodesic distances. (C) Flatten this patch to 2D using a geodesic method to preserve distances. (D) Estimate the speed and direction of the wave propagation for a subset of 20 of the recording locations. Assign the CV vector to the central measurement location. (E) Repeat for each point of the geometry to build up a spatial map of wavefront propagation velocity assuming a planar wavefront, or of longitudinal and transverse CV with ellipse orientation indicating the longitudinal fibre direction assuming an elliptical wavefront.

To reduce the dimensionality of the 3D recording locations to 2D space, a representation that best preserves the geodesic distances in the locality of the selected recordings was used; shown in Fig. 2. First of all, a subset of the mesh was taken for analysis, by selecting the vertices that are within a bounding box of the recording point subset (Fig. 2 A). The geodesic distances between all of these vertices were calculated using Dijkstra's algorithm, which finds the shortest path between vertices (Fig. 2 B). We then use the multi-dimensional scaling approach of Zigelman et al. on the matrix of geodesic distances to give 2D coordinates that best preserve geodesic distances [16,17] (Fig. 2C).

To estimate CV for the 2D locations with associated activation times, we considered each of the planar, circular and elliptical wavefront equations. CV estimates were calculated for random selections (using randperm in Matlab) of twenty recording locations together with their activation times (Fig. 2 D). In the instance that there were fewer than twenty recording locations in a region, all recordings were used for the fit. CV vectors were estimated in 2D (see Fig. 2 D, bottom left arrow) and translated to start at the mid-point of the element closest to the centre of the patch of tissue (see Fig. 2 D, central arrow). CV vectors were then projected back to the 3D geometry using barycentric coordinates to preserve the relative location within the element of the CV vector start point (centre of the element) and end points (Fig. 2 E). In this way, assuming elliptical wavefront propagation with homogeneous CV, conduction anisotropy was estimated from a single activation map by fitting to activation times measured at a set of recording locations.

### 2.4 Ellipse fitting to CV vectors from multiple activation maps (method 2)

Cardiac tissue conducts anisotropically, with faster conduction along the longitudinal fibre direction, and slower conduction transverse to the fibres. As such, CV depends on direction of propagation, with the set of possible wavefront propagation CV vectors approximately forming an ellipse [13]. We assume here that atrial tissue propagation can be approximated by a monodomain surface model, and that clinical and simulated wavefronts can be modelled with an elliptical wavefront equation. In the instance that multiple activation maps constructed from different pacing sites were available, conduction anisotropy was instead estimated by fitting an ellipse to the observed set of wavefront propagation velocity vectors (assuming planar propagation), with one vector from each activation map. In this case, we assume that wavefront curvature is sufficiently small that the speed is independent of curvature and a planar wavefront fit is appropriate.

The equation of an ellipse is given parametrically for t∈[0,2π) as:

(10) $$\left(\begin{array}{c} x\\ y \end{array}\right)=\left(\begin{array}{c} x_{0}\\ y_{0} \end{array}\right)+\left(\begin{array}{cc} \cos\;\theta & -\sin\;\theta \\ \sin\;\theta & \cos\;\theta \end{array}\right)\left(\begin{array}{c} a\;\cos\;t\\ b\;\sin\;t \end{array}\right),$$

where $\left(x_{0},y_{0}\right)$ is the known centre location corresponding to the element mid-point, *θ* is the unknown long axis orientation and *a* and *b* are the unknown long and short axis magnitudes corresponding to the longitudinal and transverse CVs respectively, giving a total of three unknowns.

We followed the method of Ray and Srivastava [18] for fitting to an ellipse. Specifically, to find the best fit ellipse to the CV vector end-points, we minimised the residuals measured along radii of the ellipse.

We first considered the case of two activation maps, in which we either fixed the ellipse orientation or anisotropy ratio, and then the case of three activation maps for which it was possible to estimate all three unknowns. In each case, the CV vectors were projected to 2D to estimate the ellipse fit, and then the calculated long axis vector direction was expressed back on the 3D geometry using a barycentric coordinate mapping.

**Fig. 3:**
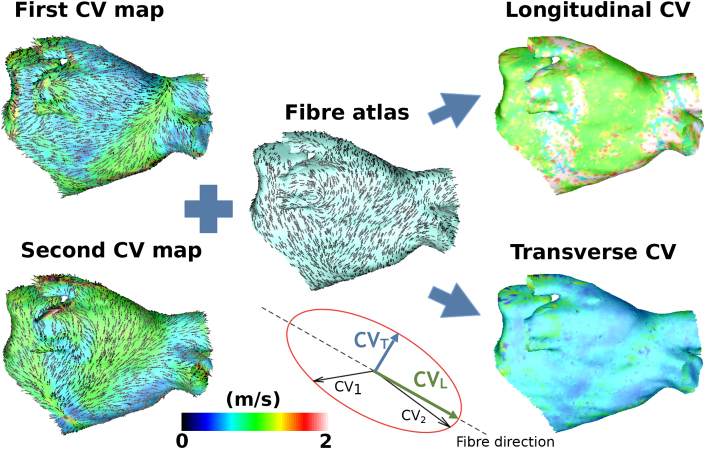
**Technique for estimating longitudinal and transverse CV from two activation maps (method 2).** Wavefront propagation velocity maps constructed from two activation maps with different pacing locations (Bachmann's bundle and the coronary sinus) are combined with a fibre atlas to estimate longitudinal and transverse CV. Planar CV is estimated for each of the activation maps; the colour indicates the CV magnitude, and the lines indicate the CV vector directions. The ellipse orientation is fixed such that the longitudinal CV runs along the fibre direction (indicated by the lines on the fibre atlas panel) and the transverse CV runs perpendicular to the fibres. The ellipse that best fits the two planar CV vectors is calculated. Longitudinal and transverse CV are then given by the magnitude of the major and minor radii respectively. The two heterogeneous wavefront propagation CV maps, calculated using the planar CV algorithm, are converted to a higher homogeneous longitudinal CV and lower transverse CV map.

For the case of fixed ellipse orientation (i.e. *θ* known in Equation (10)), we used an atlas of endocardial atrial fibres from a previously published bilayer model [19] mapped to our target geometry using our universal atrial coordinate system [20]. We assumed that endocardial activation patterns were predominantly determined by endocardial fibres. Ellipse orientation for each element was then aligned such that the long axis of the ellipse was in the longitudinal fibre direction, see Fig. 3. In this way, longitudinal CV (*a*) and transverse CV (*b*) could be estimated locally using just two CV vectors. Alternatively, we fixed the anisotropy ratio ($\frac{a}{b}$) and fitted both the ellipse orientation (*θ*) and longitudinal CV (*a*).

### 2.5 Clinical electroanatomic and imaging data

The patient studied was a 78 year old male undergoing a first clinically indicated left atrial ablation procedure for the treatment of paroxysmal AF who provided written informed consent for inclusion in the study. The study protocol was approved by a Research Ethics Committee (reference 15/LO/1803, http://www.isrctn.com/ISRCTN10910054). Cardiac magnetic resonance (CMR) imaging was acquired prior to the procedure including atrial 3D contrast enhanced gated magnetic resonance angiogram (GMRA) and 3D late gadolinium enhanced (LGE) imaging. The high contrast GMRA was segmented according to an in-house segmentation pipeline to generate a left atrial endocardial geometry [21].

Paced maps were acquired while pacing at two different locations along a decapolar catheter that was positioned in the coronary sinus (CS) throughout the procedure. Left atrial geometry was created within the Carto electro-anatomic mapping system (EAMS) (Biosense Webster, Irvine, CA) using Fast Anatomical Mapping (FAM) following respiratory training. Mapping points were acquired using a Lasso catheter (1 mm electrodes, 4 mm electrode spacing) and activation times annotated on the bipolar electrograms using the automated Carto ConfiDense module with the following settings: LAT stability - 4 ms; Catheter stability - 4 mm; point density - 1 mm. Ellipse fitting to CV vectors from two activation maps (method 2) was used to predict the longitudinal and transverse CV, assuming an atlas distribution of fibres (i.e. *θ* known in Equation (10)).

### 2.6 Simulations

To test the robustness of the algorithms in a controlled environment, we ran simulations using the monodomain tissue model and Courtemanche et al. human atrial cell model [22] on a left atrial (LA) geometry, using the CARP simulator [23]. A finite element mesh was constructed from the segmented MRI geometry by remeshing to create a more homogeneous element size of 300 *μ*m using mmgtools software (http://www.mmgtools.org/), and then fiber directions were assigned to the element mid-points of the mesh by mapping the endocardial LA fibre direction field of an atrial bilayer model [19], which we treat here as an atlas distribution. AF electrical remodelling and repolarisation heterogeneity were included in the model by modifying the cell model ionic conductances of the LA body, left atrial appendage (LAA) and pulmonary veins (PVs) as in our previous studies [24,25].

To simulate atrial pacing in the catheter laboratory, activation maps were constructed by stimulating the model in the following locations: the left superior PV (LSPV), the right superior PV (RSPV), the LAA, the proximal coronary sinus (CS), the distal CS and where Bachmann's bundle (BB) meets the LA wall. These data were used as inputs for the CV estimation algorithms (methods 1 and 2). The resulting estimates of longitudinal and transverse CV and longitudinal fibre direction were compared to the gold standard simulation values. The gold standard simulation values for the longitudinal and transverse CV were each calculated as the planar CV estimated by fitting to 100 points for a homogeneous isotropic 2D sheet simulation with either the longitudinal or transverse conductivity value. Results are expressed as the median and interquartile range (iqr) of the absolute error in CV measurements, or the error in fibre angle expressed as a value in the range [0,90). Regions of the spatial map were defined as accurate when the angle error was less than $20^{\circ }$. The accuracy of the ellipse fitting algorithms using CV vectors from two or more activation maps (method 2) depends on accuracy of the planar wavefront propagation CV estimates, which are then used to estimate the anisotropy. Areas of wavefront collision result in inaccurate planar CV estimates (with high fit residuals) because the assumption of a single wavefront breaks down. For these locations, fibre directions and CV magnitudes were interpolated from neighbouring values using Shepard interpolation.

### 2.7 Incorporating clinically measured conduction anisotropy in simulation studies

To investigate the effects of atrial conduction properties on arrhythmia dynamics, models were constructed with different conductivity properties. The first model was tuned isotropically to match the spatial distribution of longitudinal CVs, such that the effects of fibre direction and conduction anisotropy were ignored. The second model was tuned anisotropically to match local longitudinal and transverse CV estimates from the clinical data.

Similar to our previous study [26], sinus rhythm pacing was applied at the earliest activation site of the LA as reported by Lemery et al. [27] at a cycle length of 700 ms. Reentry was initiated in the model by rapidly pacing the RSPV at a cycle length of 160 ms for five beats, to model spontaneous initiation by ectopic PV triggers [28], at a coupling interval following sinus rhythm chosen depending on inducibility. To indicate the distribution of rotational activity and wavefront breakup locations in the simulation output, phase singularity (PS) density maps were calculated as in our previous publications [[24], [25], [26]]. These maps were then partitioned into low and high PS regions, which were taken to be >1 standard deviation from the mean PS value [26].

## 3 Results

### 3.1 Validation of elliptical wavefront fitting (method 1) in 2D simulations

**Fig. 4:**
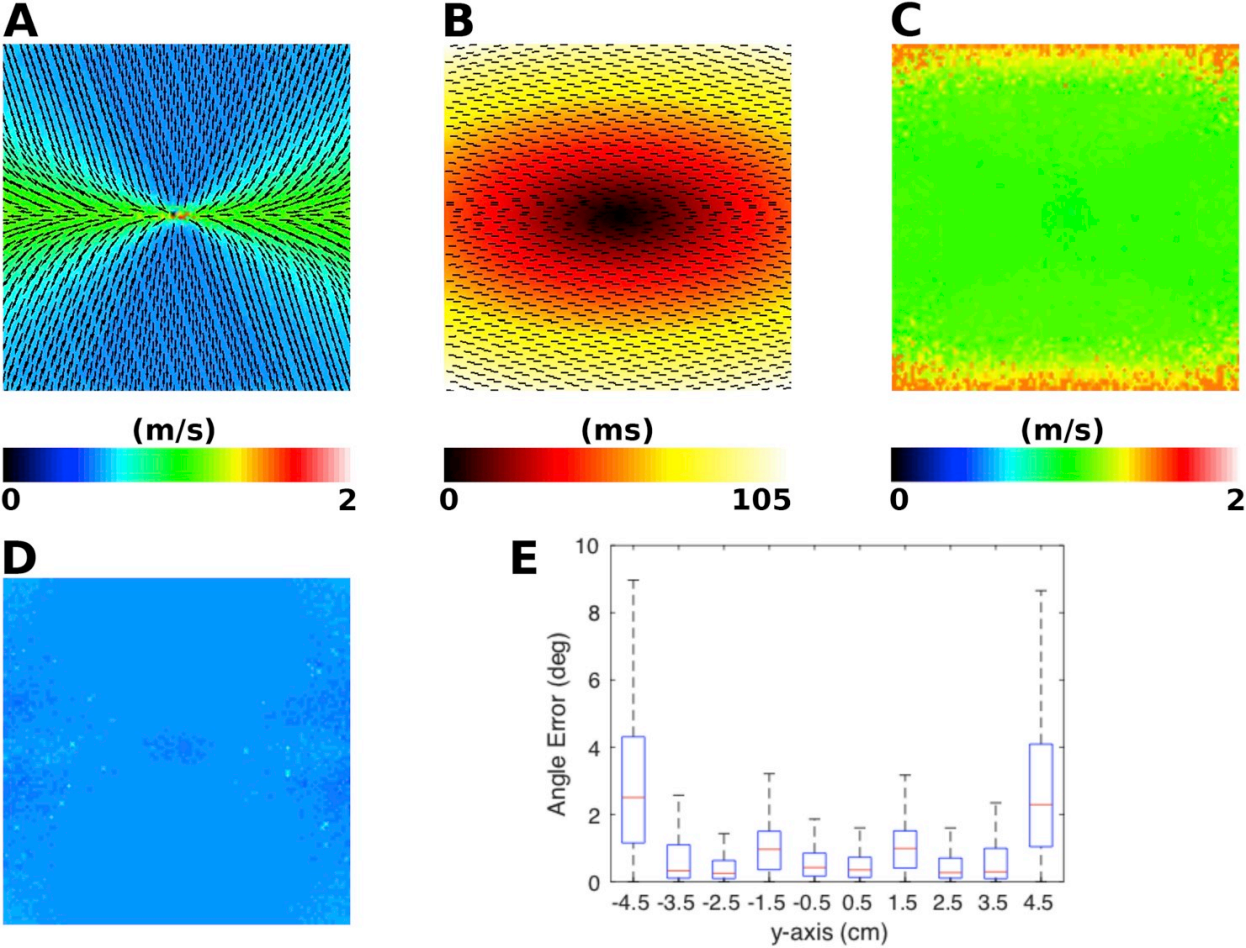
**Validation of elliptical wavefront fitting (method 1) in 2D simulations for a homogeneous anisotropic sheet.** (A) Planar CV magnitude and direction vectors (displayed as black arrows). (B) Activation time map with predicted fibre direction overlaid (displayed as black lines). (C) Estimated longitudinal CV (gold standard longitudinal CV is 1.10 m/s). (D) Estimated transverse CV (gold standard transverse CV is 0.55 m/s). (E) Box plots at 1 mm intervals showing the distribution of angle errors as a function of position along the y-axis.

The single activation map elliptical wavefront fitting algorithm (method 1, Section 2.2, Equation (8)) was first applied to data from a two-dimensional sheet simulation, with homogeneous CV and fixed anisotropy, with longitudinal fibres aligned with the *x*-axis. This is shown in Fig. 4. Wavefront propagation CV magnitude is higher when the activation is aligned with the fibre direction (see Fig. 4 (A)). Using the activation time map shown in Fig. 4 (B) to estimate the atrial fibre direction shows that the algorithm more accurately predicts the fibre direction close to the source location, indicated by the closer alignment of the estimated fibres with the *x*-axis in the centre of the domain (see Fig. 4 (E)). Longitudinal fibre estimation was accurate; median error 0.6° (iqr: 0.2–1.1°). Longitudinal CV magnitude estimation is accurate close to the pacing location (green region in Fig. 4 (C)), but overestimated further from the pacing location (red region in Fig. 4 (C)); median absolute error: 0.03 m/s (iqr: 0.01–0.06 m/s) (simulation value 1.10 m/s). The algorithm accurately estimates the transverse CV magnitude (shown by the homogeneous distribution in Fig. 4 (D)); median absolute error 0.03 m/s (iqr: 0.02–0.04 m/s) (simulation value 0.55 m/s).

### 3.2 Application of the ellipse wavefront fitting algorithm (method 1) to atrial geometry simulations

The single activation map elliptical wavefront fitting algorithm (method 1, Section 2.2, Equation (8)) was next tested on realistic atrial geometry simulations. The ability of the algorithm to accurately estimate longitudinal and transverse CV and fibre direction is quantified in Table 1 for six pacing locations. The median estimated longitudinal and transverse CV magnitudes were close to the actual values for all pacing locations (range of median values for longitudinal CV: 1.09−1.15 m/s, actual 1.10 m/s; range of median values for transverse CV: 0.54−0.60 m/s, actual 0.55 m/s). Longitudinal and transverse CV error and fibre direction angle error are relatively similar across the pacing locations. The percentage of the atrial surface area for which atrial fibres are accurately estimated is also similar (39.43−47.07%).

**Table 1:** **Single activation map ellipse wavefront fitting algorithm (method 1) results.** Simulation longitudinal CV is 1.10 m/s, transverse CV is 0.55 m/s. Accurate (%) is the percentage of the atrial area for which the angle estimation is within 20° of the input fibre direction. Errors are quoted as the median of the absolute errors and the iqr.

Pacing	Error $CV_{L}$ (m/s)	Error $CV_{T}$	Error *θ* (°)	Accurate %
BB	0.26 (0.08−0.76)	0.16 (0.06−0.29)	25.6(9.8−54.2)	42.82
CS1	0.27 (0.09−0.73)	0.15 (0.06−0.27)	24.8(9.2−54.2)	44.02
CS2	0.26 (0.08−0.79)	0.17 (0.08−0.31)	28.8 (10.7−59.9)	39.43
LAA	0.34 (0.13−0.87)	0.12 (0.04−0.24)	22.2 (8.9−47.2)	46.75
LSPV	0.33 (0.15−0.68)	0.10 (0.04−0.21)	21.7 (9.6−44.9)	47.07
RSPV	0.28 (0.11−0.69)	0.15 (0.06−0.27)	25.6 (10.4−52.6)	42.35

**Fig. 5:**
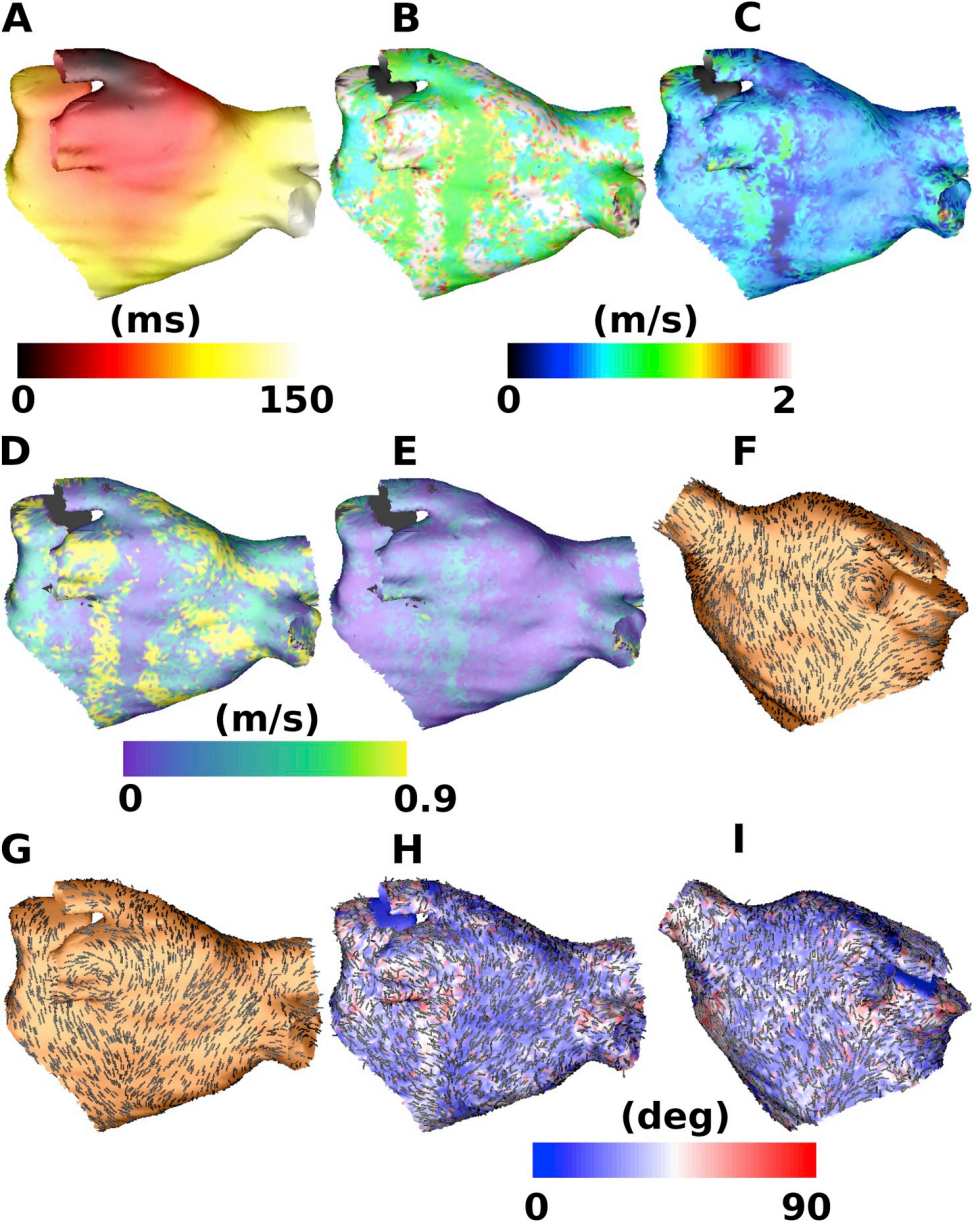
**Ellipse wavefront fitting algorithm for a single activation map (method 1) applied to atrial geometry simulations.** An example is shown here with pacing from the LSPV. (A) Activation map. (B) Longitudinal CV. (C) Transverse CV. (D) Absolute error in longitudinal CV. (E) Absolute error in transverse CV. (F) Atlas fibre map used for simulation (shown as black lines) in anteroposterior view. (G) Atlas fibre map in posteroanterior view. (H) Estimated fibre direction map coloured by angle error, with correctly estimated areas shown in blue, and incorrectly estimated areas in red for the posteroanterior view. Estimated fibre directions are displayed as black lines. (I) Equivalent map to (H) for the anteroposterior view.

The spatial distributions of angle error and longitudinal and transverse CV estimates indicate that these values are generally more accurate close to the pacing location, and less accurate further away. Changes in fibre direction between the pacing location and recording location mean that the assumption of fixed CV along a fixed longitudinal and transverse fibre axis no longer holds and estimates are less accurate. An example is shown in Fig. 5 with pacing from the LSPV (Fig. 5 A). The LSPV map is more accurate on the posterior wall and roof (B, C and H) since the pacing location is closer to these regions, and less accurate on the anterior wall (I). The longitudinal and transverse CV maps are more homogeneous than the corresponding planar wavefront propagation velocity map; however, there are regions in which the longitudinal CV is overestimated (red and white regions in B, resulting in larger errors in D). Fibre directions in the atrial body are well estimated by the algorithm (compare the atlas fibres shown in F and G and the predicted fibres in H and I); however, there are inaccuracies where there are abrupt changes in fibre direction in the input map (for example on the posterior wall below the left inferior PV, Fig. 5 G and H). As such, fibre direction and longitudinal and transverse CV cannot be accurately estimated for the entire atrial surface from a single activation map.

### 3.3 Validation of anisotropy and fibre direction estimation from multiple simulated activation maps (method 2)

The methodology developed for estimating fibre direction and anisotropy by ellipse fitting to wavefront propagation velocity vectors from multiple activation maps (method 2, Section 2.4, Equation (10)) was tested on atrial geometry simulations. The dependence of the ellipse fitting methodology on the choice of underlying activation maps was investigated by varying the activation pacing directions used for the estimation.

Table 2 shows results using two activation maps, assuming an atlas distribution of fibre directions (i.e. assuming *θ* is known in Equation (10)). Longitudinal CV magnitude estimates show a similar degree of error to the single activation map ellipse fitting method 1, whereas transverse CVs are estimated more accurately (range of median absolute errors in transverse CV for method 1: 0.10−0.17 m/s and for method 2: 0.06−0.09 m/s).

**Table 2:** **Two activation map ellipse fitting results, using an atlas of fibre directions (method 2).** Simulation longitudinal CV is 1.10 m/s, transverse CV is 0.55 m/s. Errors are quoted as the median of the absolute errors and the iqr.

Pacing	Error $CV_{L}$ (m/s)	Error $CV_{T}$ (m/s)
BB-LSPV	0.22 (0.06−0.88)	0.07 (0.03−0.15)
CS1-LSPV	0.31 (0.08−0.90)	0.06 (0.03−0.12)
CS2-BB	0.11 (0.04−0.42)	0.09 (0.04−0.18)
RSPV-LAA	0.34 (0.09−0.90)	0.06 (0.03−0.13)

For the case of assumed anisotropy ratio (i.e. assuming a/b is known in Equation 10), the longitudinal CV is estimated more accurately than using method 1 or method 2 for the case of assumed fibre direction (range of median absolute errors in longitudinal CV for method 1: 0.26−0.34 m/s and for method 2 with assumed anisotropy ratio: 0.17−0.20 m/s). However, fibre direction estimation is less accurate (method 2 with assumed anisotropy ratio: median angle error 26.9–35.0°, accurate region 30.0–39.7 %).

**Fig. 6:**
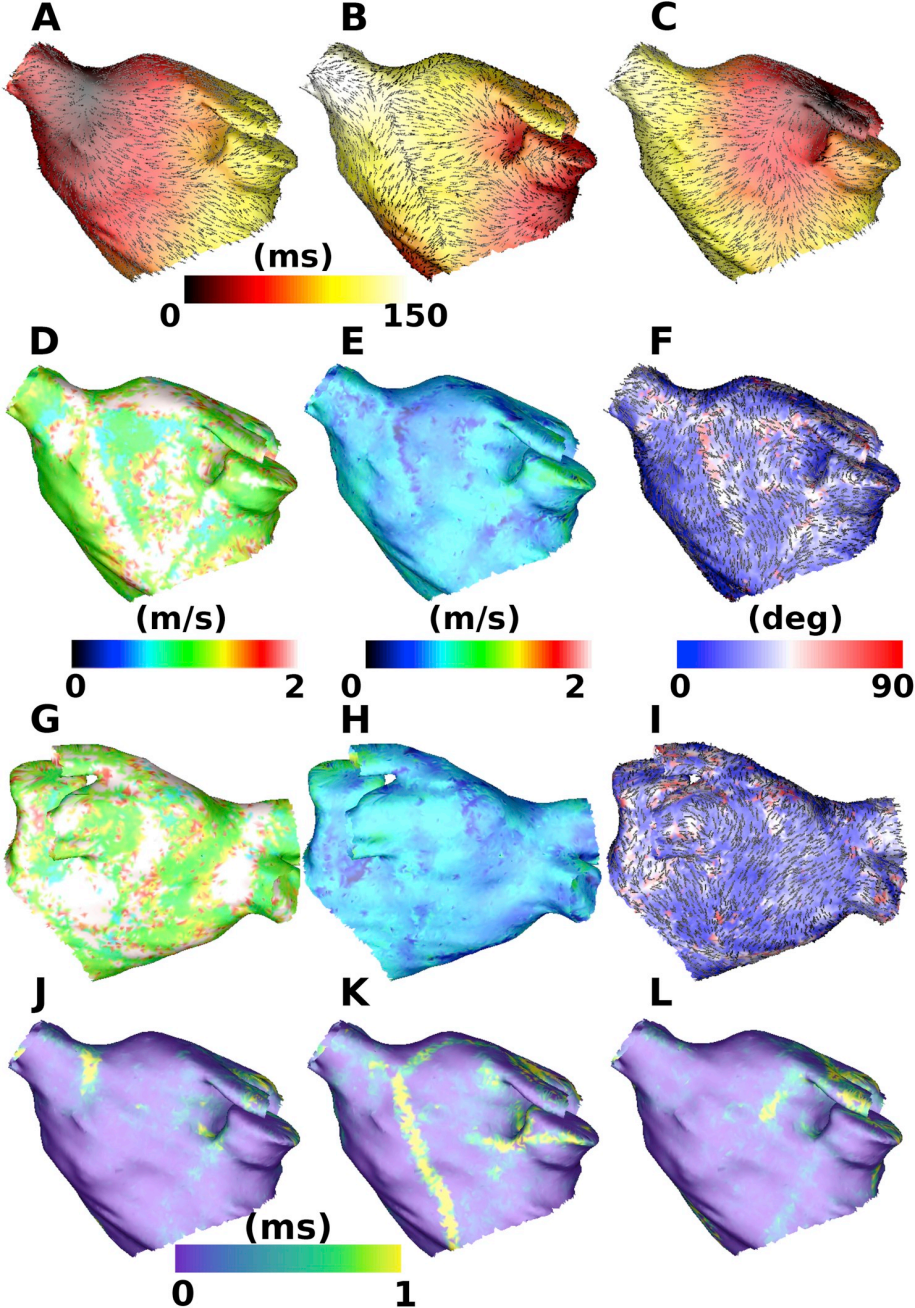
**Estimating anisotropy and fibre direction by ellipse fitting to planar wavefront propagation CV estimates from three simulated activation maps.** Activation maps with planar wavefront propagation CV vectors overlaid (black arrows) from pacing at (A) BB, (B) proximal CS, (C) LSPV. Anteroposterior views for (D) longitudinal CV map, (E) transverse CV map, (F) estimated fibre direction map coloured by fibre angle error (estimated fibre directions are displayed as black lines). Posteroanterior views for (G) longitudinal CV map, (H) transverse CV map, (I) estimated fibre direction map coloured by fibre angle error. Average residuals for pacing at (J) BB, (K) proximal CS, (L) LSPV.

The algorithm was next applied to simulations with three activation maps from different pacing directions (method 2, Section 2.4, Equation (10)). Fig. 6 shows an example set-up in which the fibre direction estimation and longitudinal and transverse CV estimation are visually better than the results of applying the ellipse fit to a single activation map (method 1). For all three combinations of pacing directions tested, there are areas of the map in which angle estimation is inaccurate; however, these regions are much smaller than the cases using one or two pacing directions. These areas are typically in the vicinity of wavefront collision in one of the underlying activation maps (for example the collision region ranging from the RSPV to the MV in Fig. 6 B is seen as an area of high angle errors in Fig. 6 F), or are regions where the three activation maps display similar CV vector directions. Regions corresponding to wavefront collision in one of the planar maps are excluded from the ellipse fitting algorithm by employing a threshold on the planar fit residual, since the planar CV vectors are inaccurate in this case (the assumption of a single wavefront breaks down, see yellow regions in Fig. 6J–L), and replaced by interpolated vectors, which introduces error (compare Fig. 6(F) with J-L).

The accuracy of the three activation map method is quantified in Table 3. Transverse CV is estimated with increased accuracy compared to the previous methodologies, and approximately 70% of fibre directions are estimated accurately for this method, compared to 30−50% for the cases with one or two pacing directions (compare Table 1, Table 2, Table 3). To test the effects of the planar wavefront approximation on the estimated CV values used as the input to method 2, we compared planar and circular CV estimates. CVs for the planar and circular fits were similar: mean absolute difference in planar and circular CVs: 0.03±0.07 m/s; 5.9% of points with CV difference > 0.1 m/s.

**Table 3:** **Three activation map ellipse fitting algorithm (method 2) results from testing on simulated data.** Simulation longitudinal CV is 1.10 m/s, transverse CV is 0.55 m/s. Accurate (%) is the percentage of the atrial area for which the angle estimation is within 20° of the input fibre direction. Errors are quoted as the median of the absolute errors and the iqr.

Pacing	Error $CV_{L}$ (m/s)	Error $CV_{T}$	Error *θ* (°)	Accurate %
CS1-LSPV-BB	0.17 (0.06−0.76)	0.06 (0.03−0.11)	11.8 (5.26−24.9)	67.74
RSPV-CS2-LAA	0.17 (0.06−0.86)	0.06 (0.03−0.11)	10.7 (4.92−21.5)	72.33
BB-LAA-LSPV	0.19 (0.06−0.88)	0.06 (0.02−0.11)	12.1 (5.40−24.8)	68.09

### 3.4 Effects of errors in assumed fibre field on longitudinal and transverse CV estimation

Ellipse fitting to wavefront propagation velocity vectors using two activation maps (method 2, Section 2.4, Equation (10)) requires an additional assumption to fit the three unknown parameters; for example, *θ* may be assumed from an atlas. Since patient-specific atrial fibres are not known, and an individual patient may show large deviations from any given fibre atlas, we investigated the effects of deviations in the assumed fibre atlas from the actual fibre directions on estimated longitudinal and transverse CV using simulation. The results of adding Gaussian error terms to fibre direction are shown in Table 4. The error terms are independent identically distributed normal error terms of a given standard deviation, and the resulting median absolute error in both longitudinal and transverse CV increases with increased perturbation in fibre direction.

**Table 4:** The effects of random errors in the assumed fibre atlas on longitudinal and transverse CV estimation.

Angle perturbation sd (deg)	Error $CV_{L}$ (m/s)	Error $CV_{T}$ (m/s)
0	0.11 (0.04−0.42)	0.09 (0.04−0.19)
14.3	0.17 (0.05−0.44)	0.11 (0.05−0.24)
28.6	0.22 (0.08−0.43)	0.16 (0.07−0.29)
43.0	0.24 (0.10−0.41)	0.20 (0.08−0.32)
57.3	0.25 (0.12−0.40)	0.22 (0.10−0.33)

### 3.5 Clinical example using method 2 with an assumed fibre atlas

**Fig. 7:**
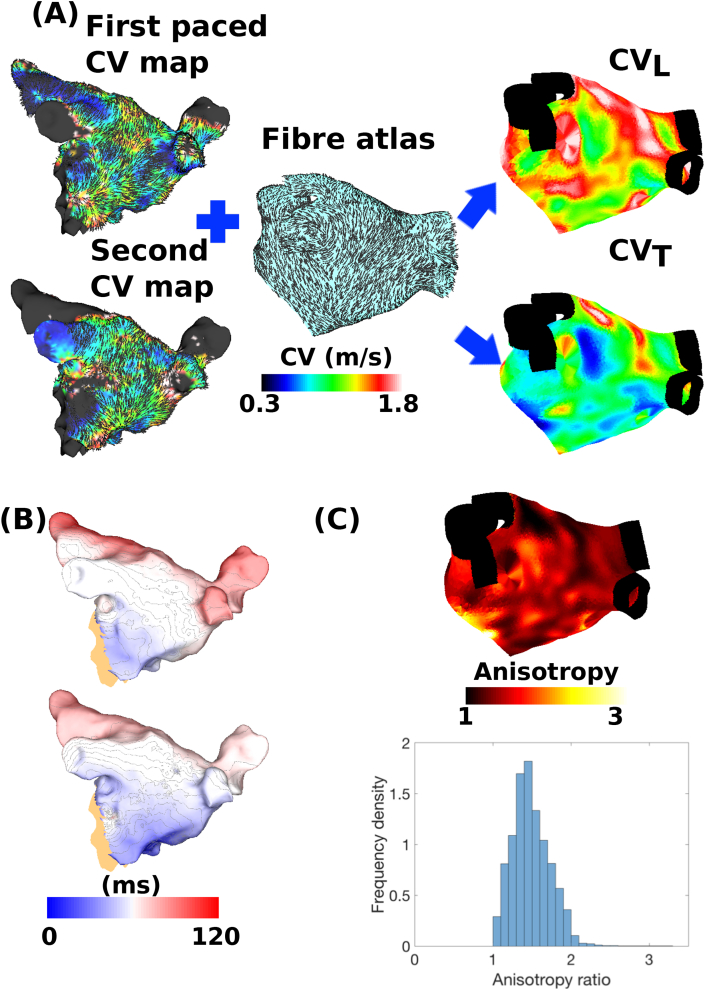
**The left atrium clinically exhibits heterogeneous anisotropy in CV.** (A) Calculation of longitudinal and transverse CV maps. Two CV maps were constructed by assuming planar wavefront propagation for activation maps paced from the proximal and distal CS. Ellipse orientation was fixed using an atrial fibre atlas, and then the long and short axis magnitudes were estimated by fitting to the two local CV vectors to estimate longitudinal and transverse CV (method 2 with *θ* fixed). Black arrows for the CV maps indicate planar CV direction, and black lines for the fibre atlas indicate the fibre direction. Black regions of the mesh are not included in the calculations. (B) Activation time maps corresponding to the CV maps given in A. Orange regions of the mesh indicate excluded areas. (C) Spatial anisotropy map and histogram of anisotropy ratios, with mean 1.49±0.24.

Fig. 7 shows the results of estimating anisotropy by applying the ellipse fitting algorithm to wavefront propagation velocity vectors from two clinically recorded activation maps, assuming an atlas distribution of fibre directions (method 2, Section 2.4, Equation (10), with *θ* from an atlas). CV is seen to exhibit anisotropy since the estimated longitudinal CV values are higher than the transverse values. The distribution of longitudinal and transverse CV are spatially heterogeneous, and the ratio between them is also heterogeneous (for example both are high on the LA roof, whereas there are larger differences between them on the posterior wall). This is quantified as a histogram of anisotropy values in Fig. 7(C), in which a large range of values is observed, with a mean of 1.49±0.24.

### 3.6 Simulating heterogeneous CV and heterogeneous anisotropy

**Fig. 8:**
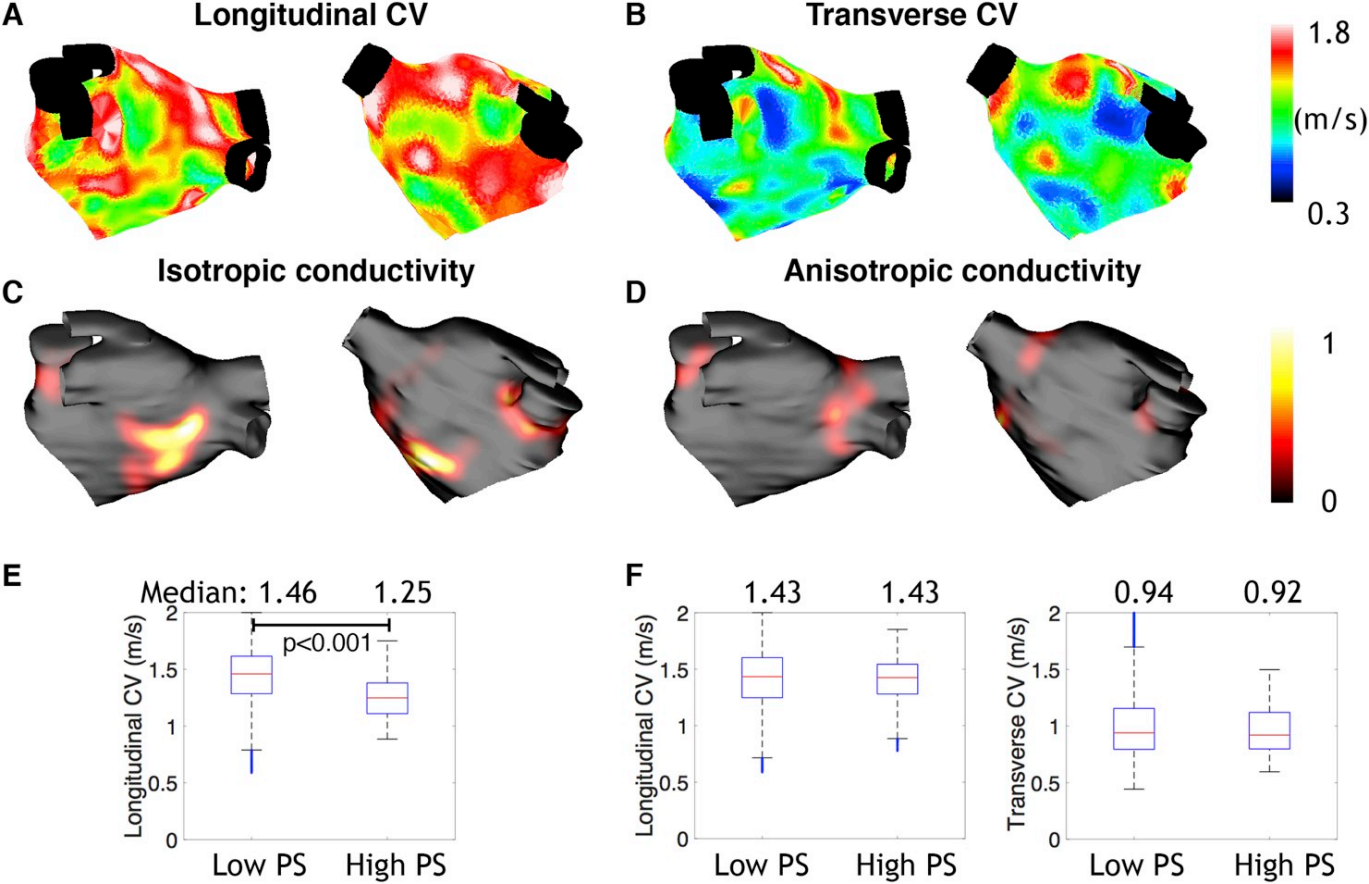
**Heterogeneous CV and heterogenous anisotropy modelling removes any direct effect of CV on PS location in simulation studies.** (A) Longitudinal CV (posteroanterior and anteroposterior views). (B) Transverse CV. Black regions of the mesh are not included in the calculations. Phase singularity density maps for isotropic conductivity in (C) and anisotropic conductivity in (D). Mean number of PSs is 1.45 for the isotropic case and 1.28 for the anisotropic case. (E) Box and whisker plots showing the longitudinal CV values in low and high phase singularity (PS) regions for the isotropic conductivity simulations. (F) Box and whisker plots showing the longitudinal CV and transverse CV values in low and high phase singularity (PS) regions for the anisotropic conductivity simulations.

The effects of calibrating CV and its anisotropy by matching local longitudinal and transverse CV measurements (a heterogeneous CV field with heterogeneous anisotropy) was compared to tuning to longitudinal CV alone (an isotropic heterogeneous CV field). These models differ in their reentry dynamics and rotor locations, as shown in Fig. 8. This is quantified in the box and whisker plots in Fig. 8, in which PSs anchor to regions of low longitudinal CV for the isotropic case; that is, median longitudinal CV in high phase singularity (PS) regions is significantly lower than in low PS regions (1.25 m/s in high PS regions vs 1.46 m/s in low PS regions, p < 0.001 by two-sided Wilcoxon rank sum test). However, for the anisotropic case, this association is lost and the longitudinal and transverse CV do not change between low and high PS regions (see Fig. 8 F). As such, anisotropy plays a significant role and removes any direct effect of CV on PS location.

## 4 Discussion

### 4.1 Main findings

We have developed and tested automated algorithms for estimating cardiac conduction anisotropy, including longitudinal and transverse CV and fibre direction, from distributions of recording locations. The algorithms work for any arrangement of points on the atrial surface and for any pacing location. The first algorithm is applied to a single activation map by fitting to elliptical wavefront propagation (method 1), which works well close to the pacing location; however, it decreases in accuracy further from the pacing location where the assumption of homogeneous conduction anisotropy breaks down. As such, we developed a methodology for measuring local conduction anisotropy – to capture heterogeneities in fibre direction – and longitudinal and transverse CV using data from two or three activation maps (method 2). The use of two activation maps requires an additional assumption: either that the atrial fibres follow an atlas distribution of fibres, or that the anisotropy ratio is known. Ellipse fitting to CV vectors from two activation maps leads to an improved estimation of longitudinal and transverse CV compared to the single activation map elliptical wavefront technique (method 1), but fibre direction estimation is still relatively poor. Using three activation maps (method 2) to estimate the longitudinal and transverse CV and atrial fibre direction provides a more accurate estimation, with approximately 70% of atrial fibres estimated within 20°. We applied the technique to clinical activation maps to demonstrate the presence of heterogeneous conduction anisotropy, and then tested the effects of this conduction anisotropy on predicted arrhythmia dynamics using computational simulation. These techniques may be applied to measure the direction dependency of atrial activation, without the need for specialised pacing protocols, in order to relate conduction anisotropy to other clinical variables.

### 4.2 Algorithm validation

The single activation map elliptical wavefront fitting algorithm (method 1) extends our previous planar and circular fitting algorithms [15] by assuming an elliptical propagation wavefront to provide an estimate of both longitudinal and transverse CV and fibre direction. The elliptical wavefront fit is derived from our circular wavefront fit by applying a linear transformation to convert elliptical wavefronts to circular wavefronts (see Fig. 1). The technique requires a minimum of five recording points per fit, compared to the requirement of three for the planar wavefront and four for the circular wavefront, which increases resolution requirements, or equivalently decreases measurement locality. This technique assumes homogeneous propagation of the elliptical wavefront between the source location and recording points, which requires a homogeneous fibre field with homogeneous longitudinal and transverse CV. As such, the technique decreases in accuracy with increased distance from the source location, since underlying fibre fields are typically heterogeneous (see Fig. 5). To measure heterogeneities in the fibre field, a second methodology was developed that estimates local conduction anisotropy by ellipse fitting to local CV vectors measured from multiple activation maps (method 2). As such, method 2 has greater data requirements since multiple activation maps are required, but it likely has the advantage that it provides a more local measure.

For two activation maps (method 2), a further assumption is required to fit the three unknowns (Equation (10)), and the choice of whether to assume an atlas distribution of fibres (*θ*) or a known anisotropy ratio (a/b) depends on a balance between which is known with more certainty and which is the more desirable measurable. Our sensitivity testing showed that estimation is sensitive to the assumed anisotropy ratio, the atlas distribution of fibres and the measurement area used for the CV estimation. The optimal region size will depend on the wavelength of the underlying propagation [29], the tissue depth, the point density, and the recording device.

The single activation map technique (method 1) showed similar overall accuracy across pacing locations, but each estimation was more accurate in the locality of the pacing location, which should be considered when selecting pacing locations. The multiple activation map technique (method 2) was sensitive to both areas of wavefront collision in the activation maps and may also decrease in accuracy in areas where CV vectors across each map are close to collinear; as such, pacing locations should be chosen as sites that are as close to orthogonal as possible. The combinations tested in this study show similar overall accuracy, and importantly three maps characterise a large proportion of the atrial surface, without the need for special pacing protocols for each atrial region. Method 2 with three activation maps was far superior to both method 1 and method 2 with two activation maps, suggesting these additional data requirements may be justified in studies investigating anisotropy. However, method 2 with three activation maps is more computationally intensive; run times are as follows for Matlab running on a MacBook Pro: method 1 with 500 points: 3 min; method 1 with 5000 points: 28 min; method 2 (2 maps), 500 points: 7 min; method 2 (2 maps), 5000 points: 66 min; method 2 (3 maps), 500 points: 10 min; method 2 (3 maps), 5000 points: 94 min.

### 4.3 Comparison to other methodologies

Our methodology for calculating CV assuming either planar, circular or elliptical wavefront propagation is a cosine-fit type algorithm, which extends the work of Weber et al. [30] and Roney et al. [15] to work for different types of wavefronts and any arrangement of points on a curved surface. We incorporated surface curvature into our CV estimation in a similar way to Verma et al. [8]. The elliptical wavefront formulation extends the circular wavefront algorithm to give an estimate of conduction anisotropy, as well as source location. Mazeh et al. [31] derived an analytic expression for CV and curvature from four recording points on either a square or a circle. Their algorithm is similar to our circular wave fitting algorithm (Section 2.1), which generalises to any arrangement of measuring points [15]. Several previous studies use triangulation methods, which have the advantage that they provide a very local measure of CV, but are also sensitive to noise in activation times [4,6] and assume planar propagation. Our methodology works for any arrangement of recording points, rather than requiring a regular grid of recordings [32], and it is fully automated. One major disadvantage of our method is that it assumes a single wavefront underlying the recording points. The fit residual indicates whether this is a suitable assumption, and the data may be divided into separate wavefronts as a pre-processing step. Both polynomial surface fitting [33] and radial basis function methodologies [34] are suitable for multiple wavefronts and any arrangement of points, but have larger data requirements and may over interpolate data. Kay and Gray developed a method for estimating wavefront curvature from isopotential lines, which they demonstrate is accurate for high resolution optical mapping data [35]. Their technique could be extended to estimate curvature from lines of constant phase or normalised unipolar electrogram voltage, but it has larger data requirements than our methodology.

Linnenbank et al. [13] also measured longitudinal and transverse CV from a single activation map, where they investigate the effects of grid size on their methodology. Our methodology offers an extension to their method by automatically selecting the longitudinal direction, which is necessary for processing large quantities of clinical data. Post-processing of the CV vector fields to calculate the divergence and curl operators may allow further characterisation of underlying activation patterns, including identification of electrical sources and reentrant activity [36]. Our techniques could be applied to activation times assigned using other methodologies, or from phase mapping [37]. Other studies have investigated the relationship between voltage and CV with fibre direction [38].

### 4.4 Clinical conduction exhibits heterogeneous anisotropy

We provide a proof of principle example, which shows that the methodology may be applied to clinical data to measure heterogeneous longitudinal and transverse CV across the LA (see Fig. 7). Heterogeneities in these measurements may be important in determining critical sites that sustain AF, and measuring the directional dependency of atrial conduction may show improved correlations with measures of structural remodelling. For example, Krul et al. measured a slowing of transverse conduction but not longitudinal with increased fibrosis in the left atrial appendage [12], and Angel et al. demonstrated diverse fibrosis architecture with decreased transverse CV in goats [39]. Considering transverse CV and longitudinal CV separately may improve correlations with atrial fibrosis [9]. The effects of the number of activation maps on the estimated atrial fibre direction from clinical data will be investigated in future studies.

### 4.5 Anisotropy affects rotor location

Our simulation studies (see Fig. 8) demonstrate the importance of conduction anisotropy in determining arrhythmia dynamics. In particular, simulated rotor location changes when both longitudinal and transverse conductivity values are tuned, and the association between rotor location and low CV seen in the isotropic case is no longer seen in the anisotropic case. This is an important consideration when constructing patient-specific models.

### 4.6 Determining atrial fibre direction

It is not possible to measure atrial fibre directions globally in vivo using current imaging technologies. Previous studies have applied diffusion tensor (DT)-MRI to small sections of atrial tissue, for example the sino-atrial node [40], to the whole atria ex-vivo [41], or have used micro-CT [42] or contrast-enhancement MRI [43] to construct myofibre orientation. The high resolution atrial DT-MRI study of Pashakhanloo et al. [41] demonstrates inter-patient variability, while simulation and experimental studies suggest that atrial fibre directions may affect arrhythmia dynamics and the outcome of ablation strategies [25,44,45]. As such, inferring patient-specific atrial fibre directions is important. Our methodology may be used to estimate a functional fibre atlas in individual patients. The relationship between this fibre field and structural remodelling indicated by late-gadolinium enhancement MRI data could then be studied to investigate the interplay between fibre disarray and changes in longitudinal and transverse CV.

### 4.7 Limitations

The techniques developed here are not fully three-dimensional, but rather work on cardiac surfaces, and as such transmural propagation is not accounted for. Typically clinical measurements are either endocardial or epicardial and transmural recordings are not available; however, our algorithms could be extended to three dimensions in the case that transmural recordings are available. A second significant limitation of our approach is that areas of wavefront collision are excluded from the ellipse fitting algorithm and fibres are interpolated in these regions, introducing significant error in the instance of a locally heterogeneous fibre field. The planar wavefront fitting algorithm used to estimate the CV vectors assumes a single wavefront of activation, but this assumption breaks down in the case of wavefront collision. Adapting the planar algorithm to fit to the two wavefronts separately would overcome this limitation and allow more accurate estimation of a greater proportion of atrial fibres for a given set of activation map inputs. Planar CV estimates will be inaccurate for wavefronts with high curvature; for example in cases of focal propagation, or due to fibrosis or discrete changes in fibre direction [2]. The planar algorithm exhibits increased inaccuracy in CV estimation at the edges of a simulated domain or tissue. In addition, spatial inaccuracies due to respiratory related motion, and temporal inaccuracies in local activation time assignment in the case of fractionated electrograms will lead to inaccuracy and a degree of uncertainty in the CV assignment [46]. Fibre direction estimation was only 70 % accurate for the three activation map case. Furthermore, we did not consider changes in curvature and speed that may occur at the edge of a bath in the bidomain model [47].

We only considered one, two or three activation maps as it is unlikely that more than three activation maps with different pacing directions would be clinically available. The fibre atlas used in this study for estimating longitudinal and transverse CV from two activation maps was from a previously published rule-based approach based on histological descriptions, but other fibre fields may be more appropriate [41,48]. We did not consider two layers with different fibre direction and so did not investigate the combined contribution of epicardial and endocardial fibres, but rather assumed that endocardial activation patterns were predominantly determined by endocardial fibres.

### 4.8 Conclusions

Overall, we have developed a technique that may be used to estimate conduction anisotropy and fibre direction from clinically available electrical recordings. The proposed algorithm is not limited to atrial data, but is also applicable to ventricular data in the instance that transmural activation is not considered. Our methodology will be used for estimating patient-specific fibre distributions and conduction anisotropy, which may be used to tune computational models and to investigate the correlations between these features and structural remodelling, electrogram features and re-entry properties.
